# Supplementary material for: Social media engagement and cryptocurrency performance
Source: PLoS One. 2023 May 11;18(5):e0284501. doi: 10.1371/journal.pone.0284501 (PMC10174546; doi:10.1371/journal.pone.0284501)
Supplement: S1 Appendix — (PDF) [file pone.0284501.s002.pdf]

S1 Appendix. Derivation of Maximum Likelihood Estimates.

The log-likelihood of the dataset is

$$L(\alpha, \beta|\mathbf{n}) = -\sum_{c\in\mathcal{C}} \alpha_c v_c \left(\sum_{i\in\mathcal{I}} \beta_i\right) + \sum_{c\in\mathcal{C}} n_c \log(\alpha_c) + \sum_{i\in\mathcal{I}} l_i \log(\beta_i) + C.$$

To obtain the ML solution with set the gradient of the log-likelihood to zero, giving

$$\frac{\partial L}{\partial \beta_i} = -\sum_{c\in\mathcal{C}} \alpha_c v_c + \frac{l_i}{\beta_i} = 0 \tag{3}$$

and

$$\frac{\partial L}{\partial \alpha_c} = -v_c \beta + \frac{n_c}{\alpha_c} = 0, \tag{4}$$

where we have defined $\beta = \sum_{i\in\mathcal{I}} \beta_i$. We fix $\beta_1 = 1$, which from equation (3) gives

$$\sum_{c\in\mathcal{C}} \alpha_c v_c = l_1$$

Substituting this again into equation (3) gives

$$\beta_i = \frac{l_i}{l_1}, \quad i \in \mathcal{I}.$$

Substituting this into equation (4) gives

$$\begin{aligned} \alpha_c &= \frac{n_c l_1}{v_c \sum_{i\in\mathcal{I}} l_i} \\ &= \frac{n_c l_1}{v_c n}, \end{aligned}$$

where we have used the definition $n = \sum_{i\in\mathcal{I}} l_i$.
