# Supplementary material for: Social media engagement and cryptocurrency performance
Source: PLoS One. 2023 May 11;18(5):e0284501. doi: 10.1371/journal.pone.0284501 (PMC10174546; doi:10.1371/journal.pone.0284501)
Supplement: S1 Table — (PDF) [file pone.0284501.s003.pdf]

S1 Table. Cryptocurrency Data.

Tables 3, 4, and 5 provide more details about the cryptocurrencies used in our study, including their ticker, type of initial offering, fundraising goals, and dates of the initial offering. We also provide the number of tweets collected for each cryptocurrency and the collection dates. The tweets are collected from the beginning of the initial offering up to 30 days later.

**Ticker**	**Initial Offering Type**	**Goal (USD)**	**Initial Offering Dates**	**Tweet Count**
MATIC	IEO	5,000,000	2019-04-18 to 2019-04-25	9,487
PERL	IEO	6,700,000	2019-08-18 to 2019-08-25	9,541
BAND	IEO	5,850,000	2019-09-10 to 2019-09-17	35,813
TROY	IEO	4,000,000	2019-11-27 to 2019-12-04	9,538
WRX	IEO	2,000,000	2020-01-27 to 2020-02-04	35,784
CTSI	IEO	1,500,000	2020-04-16 to 2020-04-22	35,689
ALPHA	IEO	2,000,000	2020-10-02 to 2020-10-10	9,621
INJ	IEO	3,600,000	2020-10-10 to 2020-10-20	36,127
AXS	IEO	2,970,000	2020-10-25 to 2020-11-04	35,829

Table 3. Cryptocurrencies created before 2021 in our dataset, their initial offering type, target fund-raising goal, initial offering dates, and total tweets collected.

Ticker	Initial Offering Type	Goal (USD)	Initial Offering Dates	Tweet Count
STC	ICO	21,000,000	2021-02-01 to 2021-04-30	35,689
SFP	IEO	5,000,000	2021-02-02 to 2021-02-08	35,782
ACM	IEO	1,000,000	2021-02-17 to 2021-02-24	9,539
RAMEN	ICO	1,250,000	2021-02-19 to 2021-02-19	420
MASK	ITO	2,700,000	2021-02-22 to 2021-02-24	35,669
ARC	ICO	7,800,000	2021-03-03 to 2021-03-09	9,452
SAFEMOON	ICO	10,000,000	2021-03-06 to 2021-03-06	58,411
ETHA	IDO	10,000,000	2021-03-04 to 2021-03-04	9,441
BELT	IDO	3,000,000	2021-03-10 to 2021-03-10	9,468
TARA	ICO	3,000,000	2021-03-11 to 2021-03-11	9,486
DEFIT	IDO	1,111,000	2021-03-14 to 2021-03-30	35,729
SAFEMARS	ICO	2,500,000	2021-03-19 to 2021-03-19	16,935
ORAO	IDO	1,000,000	2021-03-21 to 2021-03-24	9,445
VEN	IDO	7,500,000	2021-03-22 to 2021-03-24	35,773
CSPR	IEO	12,000,000	2021-03-22 to 2021-03-22	9,459
COOK	IDO	300,000,000	2021-03-30 to 2021-03-31	35,931
BOSON	IDO	4,500,000	2021-04-06 to 2021-04-07	35,808
CERE	ICO	27,800,000	2021-04-07 to 2021-04-15	35,797
XNL	Private Sale	2,300,000	2021-04-09 to 2021-04-22	35,602
FINE	IDO	3,090,000	2021-04-26 to 2021-04-27	9,612
KRYPTO	IDO	1,500,000	2021-04-30 to 2021-04-30	35,895
WEC	IEO	2,300,000	2021-05-13 to 2021-05-19	35,646
LATTE	ICO	1,000,000	2021-05-19 to 2021-05-19	207
NFT	IEO	2,399,976	2021-05-20 to 2021-05-20	36,080
HOTCROSS	IFO	2,500,000	2021-05-20 to 2021-05-20	35,696
COW	ICO	1,500,000	2021-05-25 to 2021-05-25	9,470
CFG	IEO	3,506,250	2021-05-26 to 2021-05-31	9,496
LESS	Presale	1,650,000	2021-05-27 to 2021-06-01	35,701

Table 4. Cryptocurrencies created during the first half of 2021 in our dataset, their initial offering type, target fund-raising goal, initial offering dates, and total tweets collected.

Ticker	Initial Offering Type	Goal (USD)	Initial Offering Dates	Tweet Count
SAFEBTC	ICO	2,000,000	2021-06-02 to 2021-06-05	35,882
ITGR	ICO	4,410,000	2021-06-10 to 2021-06-13	35,628
BZZ	ICO	9,933,953	2021-06-13 to 2021-06-14	35,658
HMT	IEO	50,000,000	2021-06-17 to 2021-06-22	35,619
PALG	ICO	8,000,000	2021-08-05 to 2021-09-05	35,600
TUP	ICO	8,000,000	2021-08-21 to 2021-09-05	35,712
MPT	IDO	1,000,000	2021-08-29 to 2021-08-30	35,604
DMZ	Seed Sale	1,200,000	2021-09-06 to 2021-09-07	35,723
FOREX	ICO	4,410,000	2021-09-20 to 2021-09-22	35,653
DANA	ICO	1,000,000	2021-10-28 to 2021-10-28	35,991
TEX	IDO	1,980,000	2021-11-03 to 2021-11-04	35,732
YIN	IDO	4,500,000	2021-11-03 to 2021-11-05	35,636
GOG	ICO	5,500,000	2021-11-10 to 2021-11-10	9,438

Table 5. Cryptocurrencies created during the second half of 2021 in our dataset, their initial offering type, target fund-raising goal, initial offering dates, and total tweets collected.
